# Magnetic Resonance Imaging Evidence for Human Immunodeficiency Virus Effects on Central Auditory Processing: A Review

**DOI:** 10.4172/2155-6113.1000708

**Published:** 2017-07-11

**Authors:** Yi Zhan, Jay C Buckey, Abigail M Fellows, Yuxin Shi

**Affiliations:** 1 Department of Radiology, Shanghai Public Health Clinic Center, Fudan University, Shanghai, China; 2 Geisel School of Medicine at Dartmouth, Hanover, NH, USA

**Keywords:** HIV, HAND, APD, MRI

## Abstract

New research suggests that individuals with human immunodeficiency virus (HIV) have central auditory processing deficits. To review the evidence for HIV affecting parts of the central nervous system involved in central auditory processing, we performed a systematic review of the literature. The objective was to determine whether existing studies show evidence for damage to structures associated with central auditory pathways in HIV. We searched PubMed for papers that used structural magnetic resonance imaging (MRI), diffusion tensor imaging, magnetic resonance spectroscopy or functional MRI in individuals infected with HIV. The review showed that HIV affects several areas involved in central auditory processing particularly the thalamus, internal capsule and temporal cortex. These findings support the idea that HIV can affect central auditory pathways and support the potential use of central auditory tests as a way to assess central nervous system effects of HIV.

## Introduction

Although otologic problems were common early in the HIV epidemic [1], recent studies have shown either small or no differences in peripheral hearing ability (audiometry) between HIV-infected and HIV-uninfected individuals [2–4]. Bankaitis and Keith provided a case-report of an HIV+ individual with normal peripheral auditory function who also had difficulty with speech perception, particularly with competing sentences [5]. This may indicate that central auditory system may be involved in HIV infection. Recent research found that HIV positive individuals have significant differences in word recognition scores [6] and abnormalities in auditory evoked potentials consistent with a higher rate of central auditory defects [5,7,8], compared to HIV-negative subjects, showing central auditory processing deficits (CAPD) may exist in HIV infected patients. Central auditory processing is a cognitively demanding task, therefore the findings of central auditory processing deficits may correlate with HIV-related central nervous system effects, such as HIV-associated neurocognitive disorder (HAND) [9,10].

Even with active antiretroviral therapy, HIV+ individuals continue to develop neurocognitive deficits [11] and HAND. MRI and pathological studies show evidence of glial activation and inflammation even without documented cognitive deficits [12–14]. Central auditory processing deficits in HIV+ people may have relationship with HAND or may serve as an independent sign of HIV effects on the central nervous system (CNS). There have been many MRI studies of the brain in individuals with HIV. To date, however, there has not been any direct evidence that HIV affects pathways associated with central auditory processing.

The auditory pathways in the brain include sites in the brainstem (cochlear nucleus), the midbrain (inferior colliculus), the thalamus (medial geniculate body), the internal capsule, the temporal cortex, and the corpus callosum. Also, a recent study using diffusion tensor imaging (DTI) suggested that the sites in the prefrontal cortex and anterior cingulate may also be involved in auditory processing [15].

The objective of this review was to assess the evidence in existing MR studies for HIV affecting the parts of the central nervous system involved in central auditory processing.

## Methods

We searched PubMed over the last 10 years looking for articles with the search terms MRI, HIV and brain (MRI and (HIV or AIDS) and brain)) published in English. This resulted in 775 articles. Of those, only articles directly concerned with measuring biochemical, anatomical or functional changes due to HIV were included. For example, studies examining central nervous system lymphoma, neurosyphilis or other conditions were excluded.

We focused on articles that specifically mentioned anatomic structures directly involved in central auditory processing (e.g. inferior colliculus (midbrain), medial geniculate body (thalamus), internal capsule (basal ganglia region), temporal cortex, and corpus callosum (particularly the splenium)). The articles were reviewed to assess whether the anatomic structures mentioned were reasonable to include as relevant to auditory processing. For example, multiple studies examined the basal ganglia. In some studies, procedures were used to measure particular regions within the basal ganglia (e.g. caudate or putamen). These studies were not included in the analysis since these nuclei are not universally agreed to be involved in central auditory processing. Other studies, however, selected the basal ganglia region as a region of interest. In this case, the study would be included since the region of interest would include, not just the nuclei in the basal ganglia, but also the white matter in the internal and external capsule around the basal ganglia.

**Figure 1:**
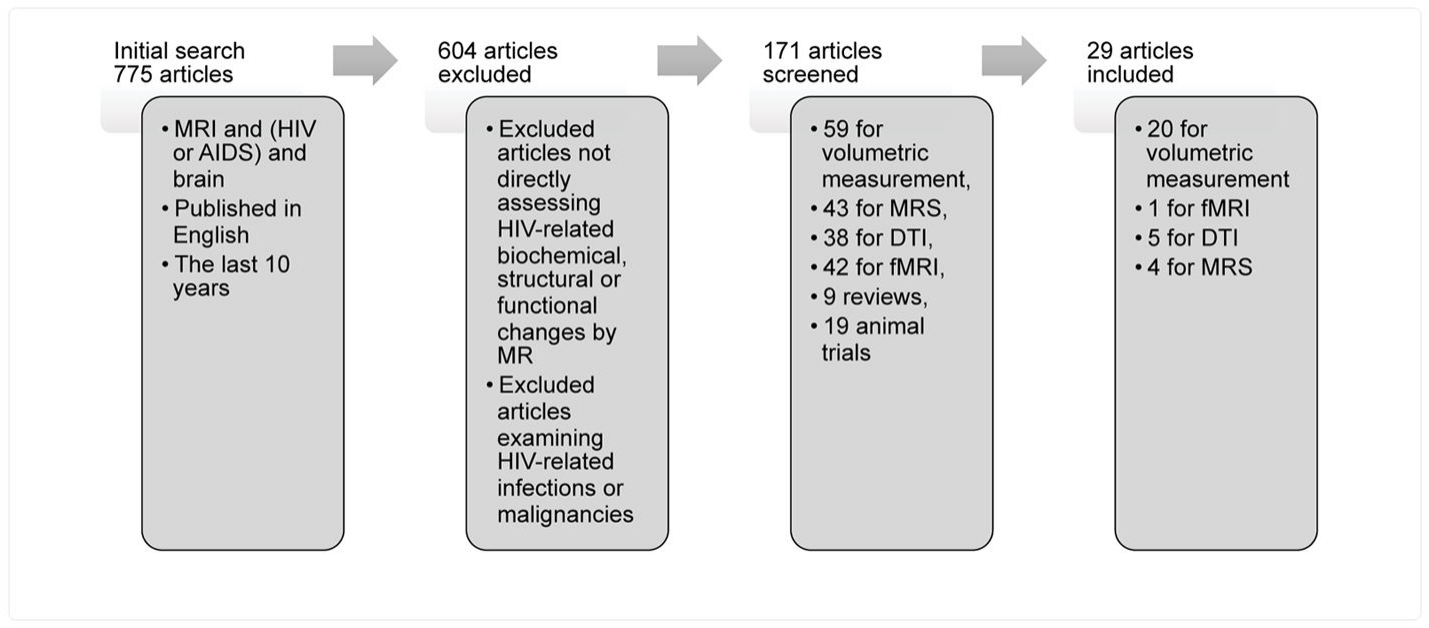
Search Strategy used to find the articles for review.

This resulted in a total of 171 articles: 59 for volumetric measurement, 43 for magnetic resonance spectroscopy (MRS), 38 for diffusion tensor imaging (DTI), 42 for functional magnetic resonance imaging (fMRI), 9 review articles, and 19 animal trials. These papers were further screened to remove articles that: (a) did not have a comparison to a control group, or were a longitudinal study within an HIV+ group, or (b) were focused on particular subsets of HIV+ people (like those with alcoholism, or taking methamphetamines). Only fMRI resting state articles were included since no articles that used an auditory stimulus were found. Figure 1 summarizes the procedure used to select the articles. The final set included 29 articles, 20 focused on volumetric measurement, 1 resting state fMRI study, 5 DTI studies and 4 MRS studies.

## Results

Compared with HIV− controls, patients with HIV have gray matter atrophy, diffuse white matter abnormalities, differences in resting state activation on fMRI, and changes in neuronal metabolites in various brain regions. Changes in brain areas relevant to central auditory processing are summarized in Table 1.

### Tissue loss and white matter abnormalities from T1 and T2 imaging

T1-weighted imaging by high resolution MR in a 1.5T or 3T field, can be used to measure the volume of brain regions. Aylward et al. [16] concluded that HIV infection causes generalized brain atrophy and also selective basal ganglia atrophy, consistent with the characterization of HIV dementia as subcortical. The internal capsule, however, was excluded from their analysis. However, some researchers studied 94 HIV+ individuals and assessed the severity of white matter abnormalities in the basal ganglia region. Thirty-two percent of the subjects (30/94) had detectable white matter abnormalities in this region.

Heaps et al. [17] found significantly smaller brain volumes in their HIV+ HAND+ group compared to the HIV− group. There was a tendency for thalamic volume to be smaller in the HIV+/HAND+ group compared to the HIV− group (p=0.06). When the analysis was done categorizing the subjects by functional impairment, thalamic volume was significantly smaller (p=0.02) in those HIV+ indiviudals with functional impairment compared to HIV− controls. Wade et al. using surface-based shape analysis in an older HIV+ cohort (mean age 65), also showed significantly smaller thalamic volume in HIV+ subjects compared to HIV− controls. Since the thalamus contains the medial geniculate body, this finding may be relevant for auditory processing.

The corpus callosum may also be affected. Wade et al. [18] showed that HIV+ individuals had a significantly smaller callosum compared to HIV− participants (p<0.05). Sarma et al. [19] found white matter atrophy in perinatally HIV-infected youths bilaterally in the posterior corpus callosum.

Overall, the studies showed both gray matter and white matter abnormalities in various regions in individuals with HIV. Areas potentially relevant to central auditory processing, such as the internal capsule, thalamus, and corpus callosum were affected.

### White matter properties

Auditory white matter radiations begin in the mesencephalon and end in the auditory primary cortex, passing information from the medial geniculate nucleus in the thalamus to temporal gyri through the internal capsule.

DTI is a form of diffusion weighted imaging (DWI) used to detect white matter structures in the brain [10,20–22]. Movement of water can be anisotropic with diffusion greater along the length of the fiber (longitudinal direction) than perpendicular to it (radial or transverse direction), as myelin may restrict diffusion. In HIV+/HAND+ people, DWI and DTI show white matter abnormalities, such as decreases in fractional anisotropy (FA), increases in mean diffusivity (MD), radial diffusivity (RD) and apparent diffusion coefficient (ADC), as well as abnormalities in axial diffusivity (AD), compared to the HIV− controls in various white matter regions [13,22–31], such as the temporal white matter and the internal capsule. Chen et al. [13] found decreases in FA, and increases in MD, AD, and RD in their population of 29 HIV+ patients compared to HIV− controls. The main areas affected relevant to auditory processing were the temporal white matter and corpus callosum. FA was significantly decreased in the temporal white matter (p<0.01) and corpus callosum (p<0.01). MD was significantly increased in the temporal white matter (p<0.05) and corpus callosum (p<0.01). The splenium of the corpus callosum was affected, which is the portion of the corpus callosum most often associated with audition. No differences were observed in the internal capsule in this study, however, for all the diffusion parameters. Xuan et al., however, did find significant decreases in FA and increases in ADC in the internal capsule in their HIV+ patients with dementia, headache or epilepsy, compared to HIV− controls [32].

Leite et al. [27] also found that HIV individuals may have differences in white matter integrity in the corpus callosum and in some regions of the corona radiata. In contrast to Chen et al. [13], however, the changes in the corpus callosum were limited to the body. These results are similar to Chang et al. [33]; they also showed differences in MD in the genu of the corpus callosum, but not in the splenium. Wu et al. [34], however, studied the corpus callosum specifically in a study of 11 HIV+ people and showed decreased FA and increased MD in the splenium of the HIV+ group compared to controls. Thurnher [35] in 2005, also found decreases in FA in the genu, splenium, and frontal white matter in 60 HIV+ adults compared to 30 HIV− controls, but the changes were only statistically significant for the genu. Xuan et al. [32] also found that mean apparent diffusion coefficient (ADC) values in HIV+ adults were significantly higher in all areas of the corpus callosum (knee, body, splenium).

Zhang and Li [24] summarized previous DTI findings in HIV+ humans in their study on SIV-infected macaque brains. They noted that FA “decreases significantly in regions such as the splenium, genu, internal capsule, frontal lobes, parietal lobes, temporal lobes, and occipital lobes in both symptomatic and asymptomatic HIV patients.” In summary, the DTI studies show that the temporal white matter, internal capsule and corpus callosum had the significant difference in FA, ADC, MD, RD in individuals with HIV compared to HIV− controls. This is significant since all these areas may be involved in central auditory processing.

### Differences in metabolism

MRS provides information on the levels of some brain chemicals that are believed to be related to glial activation and inflammation, such as choline (CHO), myo-inositol (MI), N-acetylaspartate (NAA) and creatine (Cr). The region of interest used for MRS studies, however, is large. MRS results reflect a volumetric average over a region of interest chosen in the MR image. The basal ganglia region is often selected as an area of interest in MRS studies. Unlike the volumetric studies, however, this region of interest is not specific to the basal ganglia. The region of interest likely also includes the white matter that surrounds the basal ganglia such as the external and internal capsule.

Masters and Ances reviewed the data on MRS in HIV infection in 2014. At that time they found more than 75 articles that had used MRS to detect changes in cerebral metabolites in patients with HIV. The basal ganglia region is commonly affected, and the effects of treatment in the basal ganglia region can be detected using MRS. In a recent study, Sailasuta et al. evaluated HIV individuals before and after 12 months of cART [14]. At baseline, they found elevated total CHO (p<0.002) and NAA (P<0.05) in the BG region associated with HIV infection. With treatment, inflammatory markers tended to decrease in the BG region, suggesting that the changes were HIV related [14].

Overall, these studies showed significant differences in metabolites in basal ganglia region in HIV individuals. These changes in the basal ganglia region likely also reflect the white matter tracts carrying auditory information that pass through that area.

### Neuronal activity

Resting state BOLD-fMRI can be used to assess functional connectivity within the brain. In a recent study, Ann HW et al. [36] collected the resting state fMRI data of 24 HIV+ individuals (12 with HIV+/HAND+ and 12 with HIV+/HAND−) and 11 HIV− individuals. The authors found significantly decreased functional connectivity in the temporal lobe in the HIV+/HAND+ group compared to the HIV+/HAND− group and in the HIV+/HAND− group compared to the HIV− group.

To date, however, the differences in fMRI responses between HIV+ and HIV− subjects using an auditory stimulus have not been assessed.

In summary, resting state fMRI study showed functional connectivity changes in the temporal area, which may support the hypothesis that HIV may affect the central auditory processing.

## Discussion

People with central auditory deficits may have neurological defects in the pathways from the auditory nerve through to the higher auditory pathways in the brain [37]. These deficits often manifest as difficulty with understanding speech in background noise despite normal auditory thresholds, and trouble with localizing sound or recognizing voices. The central auditory system includes the cochlear nucleus, trapezoid body, lateral lemniscus and superior olivary complex in the brainstem, the inferior colliculi (IC) in the midbrain, the medial geniculate nucleus (MGB) in the thalamus, the internal capsule in the basal ganglia region and the auditory cortex [38]. Also, the corpus callosum is involved. Damage to these structures can produce a central auditory processing deficits (usually manifested as a difficulty with understanding speech in background noise) and MRI studies done in HIV+ people to date suggest that areas associated with auditory processing are affected by HIV.

Recent large studies suggest that the auditory impairment in HIV patients cannot be explained by impaired peripheral hearing. Instead, HIV+ adults have shown evidence for difficulty in understanding speech in noise, and abnormal gap detection thresholds [2,4]. Since central auditory processing demands the collaboration of multiple brain areas, the central auditory deficit may correlate with HAND or cognitive deficits in HIV+ people [10].

Pathological studies show that the brain is widely affected by HIV infection, manifested microglia cell nodules, demyelination, glial cell proliferation, and neuronal injury [39–44]. In magnetic resonance imaging (MRI), these pathological changes are seen as brain atrophy, white matter abnormalities, differences in resting state activation, and metabolic abnormalities. This review shows that these changes can occur in areas associated with central auditory processing. The thalamus plays an important role in the central auditory processing and reduced thalamic volumes have been seen in HIV+ individuals. The auditory radiations pass through the posterior limb of the internal capsule and on the temporal lobe. Damage to the internal capsule has been seen both on T2 images, and can also be inferred from the MRS studies where the “basal ganglia region” was imaged. This region likely included white matter tracts carrying auditory information. Lastly, these studies also show the involvement of the temporal lobes and corpus callosum in the white and gray matter damage that occurs with HIV.

These findings may have practical relevance. To diagnose HAND, the “gold standard” is detailed neuropsychological performance testing [45]. If, however, auditory pathways are damaged with HIV infection in the brain, then it is possible that central auditory tests could be used to assess the central nervous system effects of HIV infection. These tests can be short and easy to perform, so may complement or offer an advantage over current techniques.

Based on these MR findings, it’s reasonable to assume that individuals with HIV may have central auditory processing deficits. Since HIV infection may damage central auditory pathways, central auditory tests may be useful to diagnose or track central nervous system effects of HIV. Also, it’s reasonable to assume that central auditory processing deficits will correlate to the cognitive deficits in HIV patients, which means that central auditory tests may provide a new way to assess central nervous system function in HIV+ individuals.

## Limitations

This review has limitations. There are no MRI studies in HIV+ adults that correlate evidence of central auditory deficits with MR findings. Also, a considerable amount of auditory processing can take place in the brainstem. Even though the brainstem plays a very important role in the central auditory processing, the MR studies reviewed here did not provide information on brainstem areas involved in auditory processing. The MR studies also did not focus specifically on auditory pathways. A reduction in thalamic volume, for example, does not necessarily mean that the medial geniculate body is affected. Similarly, MRS changes in the area of the basal ganglia do not definitively show that pathways carrying auditory information are damaged. Also, the results may be affected by publication bias, where the effects on HIV on the central nervous system may be overstated, since negative studies would not be published. Nevertheless, areas involved in central auditory processing were seen to be damaged using more than one imaging modality, and the number of MR studies showing these effects was large.

## Conclusion

Existing MR studies in patients with HIV show potential damage to auditory pathways using different imaging modalities. There are volume changes in auditory system relevant structures on T1 imaging, as well as white matter abnormalities on T2 imaging. DTI shows white matter changes in the corpus callosum and internal capsule. MRS shows signs of inflammation in the basal ganglia region that could be important for audition. Lastly, resting state fMRI shows differences in functional connectivity that includes the temporal lobe.

More focused studies need to be carried out using MR imaging associated with central auditory assessments. If a strong relationship between central auditory processing, HAND, and MR findings can be established, then central auditory tests could potentially be used to diagnose and track central nervous system complications of HIV infection.

## Figures and Tables

**Table 1:** Overall results from the review. Several areas associated with auditory processing show changes in patients with HIV.

Anatomic Region	Volumetric measurement for gray matter	Volumetric Measurement for White Matter/White Matter Changes	fMRI	DTI	MRS
Thalamus (medial geniculate body)	Decrease [17,30,33]				
Internal capsule (or basal ganglia region)		Decrease [33], Abnormal white matter signal [43]		FA Decrease [25,32] MD, AD, RD increase [25] ADC increase [32]	Increase: total CHO [14], NAA [14], MI, CHO/Cr [20] Decrease: glutarmate, NAA/CHO [20]
Temporal area (auditory cortex and auditory association cortex, and associated white matter)	Decrease [24,30]	Decrease [19]	Functional connectivity decreased [36]	FA decrease [13] MD, AD, RD increase [13]	
Corpus callosum		Decrease [19,20]		FA decrease [31,32,34] MD [34] ADC increase [32]	

## References

[1] Kohan D, Rothstein SG, Cohen NL (1988). Otologic disease in patients with acquired immunodeficiency syndrome. Ann Otol Rhinol Laryngol.

[2] Luque AE, Orlando MS, Leong UC, Allen PD, Guido JJ (2014). Hearing function in patients living with HIV/AIDS. Ear Hear.

[3] Torre P, Hoffman HJ, Springer G, Cox C, Young MA, Margolick JB (2015). Hearing loss among HIV-seropositive and HIV-seronegative men and women. JAMA Otolaryngol Head Neck Surg.

[4] Maro II, Moshi N, Clavier OH, MacKenzie TA, Kline-Schoder RJ (2014). Auditory impairments in HIV-infected individuals in Tanzania. Ear Hear.

[5] Bankaitis AE, Keith RW (1995). Audiological changes associated with HIV infection. Ear Nose Throat J.

[6] Luque AE, Orlando MS, Leong UC, Allen PD, Guido JJ (2014). Hearing function in patients living with HIV/AIDS. Ear Hear.

[7] Matas CG, Silva SM, de Marcon BA, Goncalves IC (2010). Electrophysiological manifestations in adults with HIV/AIDS submitted and not submitted to antiretroviral therapy. Pro-fono.

[8] Pagano MA, Cahn PE, Garau ML, Mangone CA, Figini HA (1992). Brain-stem auditory evoked potentials in human immunodeficiency virus-seropositive patients with and without acquired immunodeficiency syndrome. Arch Neurol.

[9] Reyes-Contreras L, Silva-Rojas A, Ysunza-Rivera A, Jimenez-Ruiz G, Berruecos-Villalobos P (2002). Brainstem auditory evoked response in HIV-infected patients with and without AIDS. Arch Med Res.

[10] Avison MJ, Nath A, Greene-Avison R, Schmitt FA, Greenberg RN (2004). Neuroimaging correlates of HIV-associated BBB compromise. J Neuroimmunol.

[11] Heaton RK, Clifford DB, Woods SP, Ake C, Vaida F (2010). HIV-associated neurocognitive disorders persist in the era of potent antiretroviral therapy: CHARTER study. Neurology.

[12] Françoise G, Francesco S, Ian E, Fabrice C, An S, Delphine B (1996). Neuropathology of early HIV-1 Infection. Brain Pathol.

[13] Chen Y, An H, Zhu H, Stone T, Smith JK (2009). White matter abnormalities revealed by diffusion tensor imaging in non-demented and demented HIV+ patients. NeuroImage.

[14] Sailasuta N, Ananworanich J, Lerdlum S, Sithinamsuwan P, Fletcher JL (2016). Neuronal-Glia markers by magnetic resonance spectroscopy in HIV before and after combination antiretroviral therapy. J Acquir Immune Defic Syndr.

[15] Farah R, Schmithorst VJ, Keith RW, Holland SK (2014). Altered white matter microstructure underlies listening difficulties in children suspected of auditory processing disorders: A DTI study. Brain Behav.

[16] Aylward EH, Henderer JD, McArthur JC, Brettschneider PD, Harris GJ (1993). Reduced basal ganglia volume in HIV-1-associated dementia: Results from quantitative neuroimaging. Neurology.

[17] Heaps JM, Sithinamsuwan P, Paul R, Lerdlum S, Pothisri M (2015).

[18] Wade BS, Valcour VG, Wendelken-Riegelhaupt L, Esmaeili-Firidouni P, Joshi SH (2015). Mapping abnormal subcortical brain morphometry in an elderly HIV+ cohort. Proc IEEE Int Symp Biomed Imaging.

[19] Sarma MK, Nagarajan R, Keller MA, Kumar R, Nielsen-Saines K (2014). Regional brain gray and white matter changes in perinatally HIV-infected adolescents. Neuroimage Clin.

[20] Paul RH, Laidlaw DH, Tate DF, Lee S, Hoth KF (2007). Neuropsychological and neuroimaging outcome of HIV-associated progressive multifocal leukoencephalopathy in the era of antiretroviral therapy. J Integr Neurosci.

[21] Lim KO, Helpern JA (2002). Neuropsychiatric applications of DTI - a review. NMR Biomed.

[22] Towgood KJ, Pitkanen M, Kulasegaram R, Fradera A, Kumar A (2012). Mapping the brain in younger and older asymptomatic HIV-1 men: frontal volume changes in the absence of other cortical or diffusion tensor abnormalities. Cortex.

[23] Ernst T, Chang L, Jovicich J, Ames N, Arnold S (2002). Abnormal brain activation on functional MRI in cognitively asymptomatic HIV patients. Neurology.

[24] Zhang X, Li C (2013). Quantitative MRI Measures in SIV-Infected Macaque Brains. J Clin Cell Immunol.

[25] Zhu T, Zhong J, Hu R, Tivarus M, Ekholm S (2013). Patterns of white matter injury in HIV infection after partial immune reconstitution: A DTI tract-based spatial statistics study. J neurovirol.

[26] Du H, Wu Y, Ochs R, Edelman RR, Epstein LG (2012). A comparative evaluation of quantitative neuroimaging measurements of brain status in HIV infection. Psychiatr Res.

[27] Leite SC, Correa DG, Doring TM, Kubo TT, Netto TM (2013). Diffusion tensor MRI evaluation of the corona radiata, cingulate gyri and corpus callosum in HIV patients. J Magn Reson Imaging.

[28] Ipser JC, Brown GG, Bischoff-Grethe A, Connolly CG, Ellis RJ (2015). HIV infection is associated with attenuated frontostriatal intrinsic connectivity: A preliminary study. J Int Neuropsychol Soc.

[29] Stebbins GT, Smith CA, Bartt RE, Kessler HA, Adeyemi OM (2007). HIV-associated alterations in normal-appearing white matter: a voxel-wise diffusion tensor imaging study. J Acquir Immune Defic Syndr.

[30] Pfefferbaum A, Rosenbloom MJ, Adalsteinsson E, Sullivan EV (2007). Diffusion tensor imaging with quantitative fibre tracking in HIV infection and alcoholism comorbidity: Synergistic white matter damage. Brain.

[31] Ragin AB, Wu Y, Storey P, Cohen BA, Edelman RR (2005). Diffusion tensor imaging of subcortical brain injury in patients infected with human immunodeficiency virus. J Neurovirol.

[32] Xuan A, Wang GB, Shi DP, Xu JL, Li YL (2013). Initial study of magnetic resonance diffusion tensor imaging in brain white matter of early AIDS patients. Chinese Medical Journal.

[33] Chang L, Wong V, Nakama H, Watters M, Ramones D (2008). Greater than age-related changes in brain diffusion of HIV patients after 1 year. J Neuroimmune Pharmacol.

[34] Wu Y, Storey P, Cohen BA, Epstein LG, Edelman RR (2006). Diffusion alterations in corpus callosum of patients with HIV. AJNR Am J Neuroradiol.

[35] Thurnher MM, Castillo M, Stadler A, Rieger A, Schmid B (2005). Diffusion-tensor MR imaging of the brain in human immunodeficiency virus-positive patients. AJNR Am J Neuroradiol.

[36] Ann HW, Jun S, Shin NY, Han S, Ahn JY (2016). Characteristics of resting-state functional connectivity in HIV-associated neurocognitive disorder. PLoS ONE.

[37] Rojas-Godoy AL, Gomez-Gomez O, Rivas-Munoz FA (2014). Compliance with current standards for the early detection of neonatal hearing loss. Revista de salud publica.

[38] Javad F, Warren JD, Micallef C, Thornton JS, Golay X (2014). Auditory tracts identified with combined fMRI and diffusion tractography. NeuroImage.

[39] Kaul M, Garden GA, Lipton SA (2001). Pathways to neuronal injury and apoptosis in HIV-associated dementia. Nature.

[40] Adle-Biassette H, Levy Y, Colombel M, Poron F, Natchev S (1995). Neuronal apoptosis in HIV infection in adults. Neuropathol Appl Neurobiol.

[41] Jayadev S, Garden GA (2009). Host and viral factors influencing the pathogenesis of HIV-associated neurocognitive disorders. J Neuroimmune Pharmacol.

[42] Masliah E, Ellis RJ, Mallory M, Heaton RK, Marcotte TD (1997). Dendritic injury is a pathological substrate for human immunodeficiency virus — related cognitive disorders. Ann Neurol.

[43] Steinbrink F, Evers S, Buerke B, Young P, Arendt G (2013). Cognitive impairment in HIV infection is associated with MRI and CSF pattern of neurodegeneration. Eur J Neurol.

[44] McArthur JC, Haughey N, Gartner S, Conant K, Pardo C (2003). Human immunodeficiency virus-associated dementia: An evolving disease. J Neurovirol.

[45] Ances BM, Hammoud DA (2014). Neuroimaging of HIV-associated neurocognitive disorders (HAND). Curr Opin HIV AIDS.

